# From Differential Stains to Next Generation Physiology: Chemical Probes to Visualize Bacterial Cell Structure and Physiology

**DOI:** 10.3390/molecules25214949

**Published:** 2020-10-26

**Authors:** Jonathan Hira, Md. Jalal Uddin, Marius M. Haugland, Christian S. Lentz

**Affiliations:** 1 Research Group for Host-Microbe Interactions, Department of Medical Biology and Centre for New Antibacterial Strategies (CANS), UiT—The Arctic University of Norway, 9019 Tromsø, Norway; jonathan.hira@uit.no (J.H.); jalal.uddin@uit.no (M.J.U.); 2 Department of Chemistry and Centre for New Antibacterial Strategies (CANS), UiT—The Arctic University of Norway, 9019 Tromsø, Norway; marius.m.haugland@uit.no

**Keywords:** activity-based probe, antibiotic conjugate, bacterial imaging, bacterial uptake, fluorogenic substrate, metabolic labeling, phenotypic heterogeneity

## Abstract

Chemical probes have been instrumental in microbiology since its birth as a discipline in the 19th century when chemical dyes were used to visualize structural features of bacterial cells for the first time. In this review article we will illustrate the evolving design of chemical probes in modern chemical biology and their diverse applications in bacterial imaging and phenotypic analysis. We will introduce and discuss a variety of different probe types including fluorogenic substrates and activity-based probes that visualize metabolic and specific enzyme activities, metabolic labeling strategies to visualize structural features of bacterial cells, antibiotic-based probes as well as fluorescent conjugates to probe biomolecular uptake pathways.

## 1. Introduction—From 19th Century Microbiology to Modern Day Chemical Biology

If chemical biology can be defined as the ‘interrogation of biological systems with chemical approaches’ [1], we must acknowledge some of the first microbiologists as chemical biologists. The birth of microbiology as a discipline in the 19th century microbiology was largely enabled through the implementation of non-specific chemical dyes to visualize and differentiate the causative agents of infectious diseases [2,3,4]. In 1877, Paul Ehrlich repurposed dyes that were being used in the textile industry to stain cellular structures in animal tissues and differentiate blood cells [2]. A few years later, Koch adopted one of those dyes, methylene blue, for the detection of *Mycobacterium tuberculosis* bacteria, demonstrating its causality for tuberculosis and clarifying a matter of contentious scientific debate at that time [3]. In 1884, Gram developed a differential stain, which distinguished two groups of bacteria based on their staining properties with crystal violet/iodine and a counterstain [4]. These bacteria are nowadays referred to as Gram-positive and Gram-negative bacteria. Thus, long before DNA sequence analysis enabled modern phylogenetic classification of bacterial strains, chemical stains made it possible to classify bacteria according to their staining properties in addition to morphological traits. Furthermore, as probes for the molecular architecture of the bacterial cell envelope, these differential stains already exposed structural differences in the bacterial cell envelope long before the molecular basis for the differential stain was uncovered decades later and became textbook knowledge: Gram-negative bacteria possess an outer membrane, but only a thin peptidoglycan (PG)-layer in their cell wall from where crystal violet complexes can be easily washed out by ethanol treatment, while these dye complexes are efficiently retained in the thick multilayered cell wall of Gram-positive bacteria (which do not have an outer membrane).

After identifying and differentiating the causative agents of communicable human diseases, the discovery of treatment options to kill pathogenic bacteria and cure these diseases was the largest endeavour of the field in the early-to-mid 20th century. In the second half of the 20th century the advent of molecular biology has dramatically shifted the research frontiers of microbiology: While differential stains were still carried out as a routine in medical microbiology laboratories and antibiotics had been found that promised control of most bacterial infections, the field embraced the opportunities that came with the advent of genetics and molecular biology. For imaging purposes biological tools began to outpace chemical tools in terms of versatility and specificity. Antibodies could be raised for any purified protein of choice and used for specific visualization of target localization by fluorescence microscopy and electron microscopy with unprecedented resolution. Furthermore, genetic manipulations enabled the introduction of genes encoding fluorescent reporter proteins, such as green fluorescent protein (GFP), into bacterial genomes, and study protein expression and localization in live bacterial cells in real time. Over the last two decades modern chemical biology has been established as an innovative field breaking the boundaries between chemistry and the life sciences that also empowers microbiology research.

The ‘early’ chemical probes of the 19th century, were non-specific in nature and the differential staining pattern they produced were a result of the physico-chemical properties of the dyes and the cellular structures they were retained in, mostly requiring complex procedures involving fixation and various staining, counterstaining, and destaining steps to achieve optimal contrast. In contrast, modern chemical biology provides an expanding toolset of chemical probes that allow the visualization of biomolecules in living cells and complex organisms with high-spatio temporal precision. While the development and optimization of new probes is driven forward by specialized chemical biology groups with expertise in synthetic chemistry, more traditional microbiologists are discovering the utility of this chemical toolset for biological studies.

## 2. A Guide to Structure and Mode-Of-Action of Different Chemical Probes

In order to be meaningful reporters for biological function, small molecules must have two important properties: They must be detectable and they must interact with the biological specimen in some specific way that leads to generation of contrast. Depending on the probe and application this contrast can be generated in different ways: (i) contrast between bacteria and the surrounding environment enabling bacterial detection; (ii) contrast between different bacterial cells enabling their functional differentiation; (iii) contrast between different substructures within a bacterial indicating the subcellular localization of a probe target. Before describing specific types of chemical probes and their biological applications in more detail, we will now first discuss different detection and targeting strategies that can be used in chemical probe design.

### 2.1. How to Detect and How to Target Probes

The detection of chemical probes can rely on different modalities including fluorescence, chemiluminescence, light scattering (Raman) or radioactivity, that are discussed in more detail below. Due to its versatility and ease of use, fluorescence is the most widely used modality, which we will therefore discuss in greatest detail. Fluorescence can be detected with in vitro analytical instruments (fluorescence scanners/microplate reader), by time-lapse fluorescence microscopy, super-resolution microscopy, and flow cytometry or non-invasive optical in vivo imaging chambers. As chemical probes, we will distinguish between (untargeted) fluorescent dyes [5,6] and targeted fluorescent probes [7,8,9]. A multitude of different fluorophores/fluorescent tags with different optical and physico-chemical properties are available that cater to the demands of multiplexed imaging or advanced detection methods (e.g., super-resolution imaging or in vivo imaging). Furthermore, fluorogenic probes [10,11,12,13] and quenched fluorescent probes [10,14] are activatable probes with reduced background fluorescence signals and higher specificity compared to targeted fluorescent probes, making them particularly useful for real time imaging studies. Despite this versatility, the physico-chemical properties of fluorescent tags can dramatically alter the biological activity and distribution (e.g., cellular uptake) of the biomolecule of interest. To overcome this pitfall two-step detection strategies have been developed that use minimal bio-orthogonal tag [15,16,17,18,19]. These bio-orthogonal probes are structurally more similar to their native parent biomolecules, which increases their bio-compatibility. The downsides are obvious: The more complex two-step procedures are lengthier and experimentally more challenging, which makes them in general more difficult to implement in real-time imaging applications.

Chemiluminescence is an alternative modality, which provides very high sensitivity and low background for applications ranging from in vitro analysis (microplate reader/luminescence scanning) over luminescence microscopy to non-invasive in vivo imaging [20,21,22]. In contrast to fluorescence, only a limited number of chemical scaffolds are available for the design of chemiluminescent probes, which are not compatible with flow cytometry, super-resolution microscopy or multiplexed analyses, but the field is rapidly expanding. Chemical probes for Raman spectroscopy [23,24] and radiolabeled probes for positron-emission tomography (PET)-imaging [25,26,27] have a more restricted application spectrum and will therefore only be briefly introduced.

#### 2.1.1. Chemical Dyes

The simplest type of imaging probe is constituted by chemical dyes. These molecules are inherently colored and/or fluorescent, which allows their detection by either light or fluorescence microscopy. The physico-chemical properties of the dye govern the interaction with cellular structures such as membranes or the cell wall, e.g., cell permeability. Contrast is generated by differential retention in different cellular compartments or when comparing the labeling intensity of different cells with one another. Essentially, for these dyes, the moieties that govern interaction and detection cannot be separated from each other. Examples include the common components of chemical stains such as crystal violet or methylene blue, but also simple fluorescent dyes such as nile red or propidium iodide [5,6].

#### 2.1.2. Targeted Fluorescent Probes

In contrast to simple ’fluorescent dyes’, targeted fluorescent probes consist of two conjugated moieties: (i) a targeting group that lends the probe a specific affinity to interact with certain targets or engage in certain biochemical processes; (ii) a fluorophore, which serves as a tag for detection. Similar to chemical dyes, fluorescent probes need to be enriched in certain biological sites in order to generate contrast. Targeted probes are usually based on high-affinity interactions, which is necessary to avoid non-specific interactions of the probe and background signal from free probe. Even though such probes have targeting moieties meant to determine their interaction partners and their cellular distribution and location, the size and physico-chemical properties of the fluorophore will affect parameters such as the binding affinity, non-specific interactions, or probe uptake. The availability of fluorophores with non-overlapping excitation/emission spectra allows the multiplexed detection of several fluorescent probes in one sample [11]. It should be noted that not all fluorophores are compatible with all applications. STORM-imaging, for example requires blinking fluorophores (e.g., AF647 or rhodamine-based probes) [17,28], whereas the use of fluorophores for non-invasive in vivo imaging is restricted to near-infrared dyes (such as Cy5, indocyanine green), which have a deeper tissue-penetration [29].

#### 2.1.3. Fluorogenic and Quenched Fluorescent Probes

Fluorescent probes generate a considerable amount of ´background´ signal due to the presence and non-specific interactions of ´free´ probe within the sample. This problem is addressed by fluorogenic and quenched fluorescent probes, which do not emit a detectable signal before they are activated by a specific interaction and hence produce less background. In fluorogenic probes, the detection tag is masked until the probe is activated by a specific biochemical process or interaction causing a strong increase in fluorescence. Commercially available fluorogenic substrates are most commonly based on 7-amino-4 carbamoylmethylcoumarin (ACC) or p-nitroaniline (pNA) fluorogens. A number of alternative fluorogenic reporters are emerging, such as acyloxymethyl ether fluorescein [13] or 7-hydroxy-9H-(1,3-dichloro-9,9-dimethylacridin-2-one) (DDAO)-derivatives, which emit in the far-red spectrum [30,31], or spontaneously-blinking fluorogens for super-resolution microscopy [32].

A similar principle is underlying so-called quenched fluorescent probes. Here, the probe carries two different functionalities, a fluorophore and a quencher, that are attached to different parts of the molecule. Through enzymatic cleavage of the probe, these two functionalities will be separated leading to an increase in fluorescence intensity [10].

A related detection method is based on fluorescence resonance energy transfer (FRET) where a probe accommodates a donor and an acceptor fluorophore within close proximity. In intact probes, excitation of the donor fluorophore will lead to energy transfer from donor to acceptor and a wavelength shift of the emission to the higher wavelength of the acceptor. Upon cleavage of a FRET-probe, the donor and acceptor fluorophore will become separated from each other, leading to loss of FRET-signal and gain in donor fluorescence that can be detected by ratiometric imaging [33].

#### 2.1.4. Two-Step Detection Strategies Relying on Bio-Orthogonal Tags

A drawback of fluorescent tags is their relatively large size, which can easily exceed the size of the targeting part of the molecule. Conjugation to these bulky tags may dramatically affect the affinity of the probe to native interaction partners of a targeting molecule. These considerations have led to the development of so-called bio-orthogonal labeling techniques. In a two-step process, first a probe is applied that carries a minimal tag that does not or only marginally affect the biological activities of a probe molecule. After the probe has engaged in the physiological process that it interrogates, it may be detected using a selective chemically compatible detection reagent (e.g., functionalized fluorophores) in a specific chemical reaction. This strategy is very useful, if some of the barriers or restrictions that are available for the first labeling reaction are not present anymore in subsequent detection step. A prime example for the utility of this two-step strategy is metabolic labeling strategies. Here, minimal tags are essential for probes to ensure that they are recognized as substrates by the enzymatic machinery that incorporates them into the large macromolecular scaffolds such as the cell wall, where they can subsequently be detected by bio-orthogonal chemistry. Alternatively, barriers that prevent permeability of directly fluorescently tagged probes (such as the cell wall) but are permeable to smaller probes with click-chemistry tags, may be permeabilized after labeling under physiological conditions and fixation of cells.

The most common bio-orthogonal reaction is ´copper-catalyzed azide-alkyne cycloaddition´ (CuAAC) also known as click-chemistry. The two compatible chemical handles (azide and alkyne) are very small and versatile and are widely used in in vitro studies as well as for click-reactions on fixed cells. Due to the toxicity of the Cu(I) catalyst, however, CuAAC is incompatible with use in living cells. To overcome this pitfall, alternative pairs of click-chemistry handles have been developed such as cyclooctyne/azide (strain-promoted azide-alkyne cycloaddition) [34], which specifically interact with each other under physiological conditions without the addition of catalysts. Other bio-orthogonal reactions include the Staudinger ligation [35] and tetrazine-based reactions [36]. By choosing different combinations of bio-orthogonal pairs, it is also possible to perform multiplexed labeling imaging of different molecular targets or biological processes at the same time [37].

Rapid developments in synthetic chemistry and chemical biology are driving the emergence of further additions to the bio-orthogonal chemical toolbox. Sulfur fluoride exchange (SuFEx), the ‘new generation of click chemistry’ [38], has recently progressed to applications in bioconjugation and imaging [18,39,40,41]. Other innovative approaches are enabling the introduction of traditional click chemistry handles in native proteins: for example, exposed methionine residues can be selectively functionalized with oxaziridine reagents carrying alkyne tags, thereby enabling a two-step strategy for probe incorporation [42,43].

#### 2.1.5. Chemiluminescent Probes

An emerging alternative to fluorescent probes with high sensitivity and low background signals are chemiluminescent probes. For a long time, the one dominant (bio)luminescent system in bioimaging has been enzymatic turnover of the small molecule substrate luciferin by firefly luciferase under the emission of light. However, in recent years remarkable progress has been made in making chemiluminescence decay of small molecules triggerable and detectable and therefore amenable for incorporation into small molecule chemiluminescence probes. Shabat and coworkers developed substituted phenoxy-dioxetane probes with highly increased chemiluminescence properties suitable for the chemiluminescent detection of biological activities under physiological conditions in cells or animals. In the ´dark´ probe a chemiluminescent group is masked by an analyte-responsive protective group, which can be selectively removed by reaction with the analyte of interest, generating turn-on chemiluminescence [20]. In the meantime, numerous different analyte-responsive probes have been developed to detect enzymes such as beta-galactosidase [20], esterases [44], proteases [45] and phophoesterase [44], the cancer-associated NAD(P)H:quinone oxidoreductase NQO11) [46], hydrogen peroxide [47], formaldehyde [48] or singlet oxygen [21]. For a detailed overview of the recent advances in this field the reader is referred to a review article by Hananya and Shabat [22]. In contrast to fluorescence imaging, where multiplexing is enabled through the use of multiple different fluorophores with non-overlapping excitation/emission spectra, chemiluminescent imaging does not offer this application (yet).

#### 2.1.6. Alkyne-Based Probes for Raman Spectroscopy

In addition to their utility for bio-orthogonal reactions, the small alkyne groups have a spectroscopically unique signature in the cell that can be directly imaged in live cells by stimulated Raman scattering microscopy [23]. Alkyne tag Raman imaging has now been efficiently used to study proliferation using 5-ethynyl-2′-deoxyuridine, to study cellular choline phospholipids synthesis using propargylcholine [23], to study uptake and localization of fatty acids [23,49], drugs [23,50] or sugars [51]. Alkyne-based pH sensitive Raman reporters have also been developed [52]. For a detailed overview of tags used for Raman imaging, the interested reader is referred to this recent review article [24].

#### 2.1.7. Radioactive Labels

Radioactive labels have been among the first tags used for labeling of biomolecules, but due to safety concerns and technical limitations, optical probes are usually the first choice. The one widespread application within the scope of this review article are radiolabeled probes used to detect bacteria and trace infections non-invasively by positron-emission tomography (PET)-imaging in vivo. In contrast to fluorescence-based optical in vivo imaging methods whose applicability is restricted to more superficial tissues due to the limited tissue-penetration of light, PET-imaging, which is also used in the clinics, is the modality of choice for in vivo imaging in deeper tissues. The most common probes used are metabolic probes such as ^18^F-fluorodeoxyglucose that are specifically taken up and are enriched by bacteria [53] or radiolabeled antibiotics [54]. The following review articles give further insight into recent progress and limitations of this field [54,55,56].

### 2.2. Chemical Probes to Illuminate (Micro)Biological Activities

**Figure 1:**
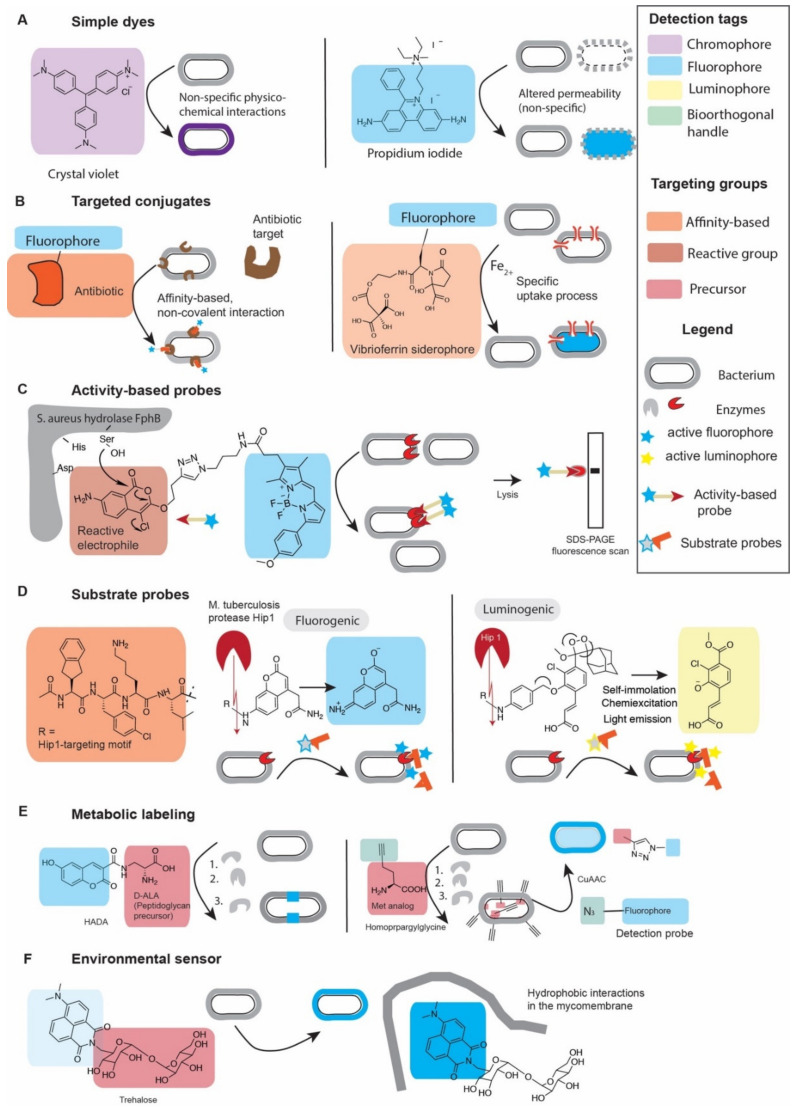
Illustrations of different types of chemical probes and their mode of action. (**A**) Simple dyes (such as crystal violet [57,58,59]) interact non-specifically with bacterial cells and stain cellular structures or are used to probe the permeability of the bacterial cell envelope (propidium iodide) [5,6]. (**B**) Targeted fluorescent conjugates as exemplified as antibiotic-conjugates and siderophore-based uptake probes [60]. (**C**) Activity-based probes are functionalized covalent enzyme inhibitors that bind irreversibly to their enzyme targets and can visualize target activity within live cells and in analytical in vitro methods. The figure shows the chloroisocoumarin-ABP JCP251-bT [7]. (**D**) Substrate probes are enzymatically turned over leading to an increase in fluorescent or chemiluminescent signals. Activity of the *M. tuberculosis* protease Hip1 can be measured using by the fluorogenic probe CSL174 [12] or the luminogenic probe FLASH [45], which are best on the same peptidic-recognintion motif. (**E**) Metabolic labeling probes are incorporated by the enzymatic machinery of the cell into cellular macromolecules. The figure showes the fluorescent D-ALA conjugate HADA and [61]) and the bio-orthogonal probe homopropargylalanine [62], which requires two-step detection with CuAAC. (**F**) The environmental sensor trehalose-DMN [63] increases in fluorescence intensity after incorporation into the hydrophobic mycomembrane.

In the following we will discuss different strategies how probes can be targeted to report on specific biological activities, which are also illustrated in Figure 1. This can be achieved by incorporation of a targeting group that enables a non-covalently high-affinity interaction between the probe and its molecular target. For enzymes, so-called activity-based probes have been developed that bind covalently to the active-site of enzymes, reporting both on the localization and activity of an enzyme. For both covalently and non-covalently target-binding probes, the contrast is generated based on the enrichment of the probe at sites where the target is localized. Alternatively, enzyme function may be studied by substrate probes that are enzymatically processed to produce a detectable signal. Another labeling strategy that makes use of functionalized substrates is metabolic labeling. In contrast to simple substrate probes, which are merely turned over by their target enzymes, metabolic labeling probes are precursors of cellular macromolecules that are incorporated by the enzymatic machinery into cellular structures such as the cell wall. Thus, metabolic labeling interrogates entire biosynthetic pathways rather than specific enzyme activities. Finally, chemical sensors produce a detectable signal upon reaction with a certain type of metabolite or upon engaging a specific molecular environment.

#### 2.2.1. Cellular Permeability and Its Effect on Specific and Non-Specific Probes

Regardless of the presence of specific intracellular targets, virtually any fluorophore of fluorophore-conjugated molecule can be used to probe cellular permeability/uptake properties (Figure 1A). Non-specific fluorescent dyes or DNA-binding dyes to probe cell permeability are among the most widely used probes in microbiology and a number of suitable dyes are commercially available. The underlying principle of differential staining is that the bacterial cell envelope can represent an impenetrable barrier to some dyes, preventing uptake and cellular labeling. However, if the biophysical properties of the cell envelope are altered or its structure is compromised, certain dyes may suddenly now enter the cells. This concept is used for example in the widely adopted live-dead stains where permeability-restricted dyes such (such as propidium iodide) can only be taken up by dead cells while a cell-permeable counterstain (e.g., Syto9) can also penetrate and label live cells. Whereas fluorescent dyes to stain have long been commercially available, new dyes have been developed for multi-color super-resolution imaging of the bacterial membrane and nucleoids [28]. It is important to note that cellular permeability is of relevance when interpreting cellular labeling with probes that have to overcome cellular barriers before engaging in specific interactions with their target. Any changes in the labeling between cells could in theory be attributed to either changes in target abundance or in altered cell permeability or in a combination of both.

#### 2.2.2. Non-Covalent Targeted Conjugates

Any type of specific molecular interaction can in theory be probed if one of the interaction partners is conjugated with a detection group (such as a fluorophore) (Figure 1B). In microbiology, most non-covalently binding chemical probes are derived from antibiotics and in practice, their functionalization almost always leads to a compromise in their biological activity. Nevertheless, many fluorescent antibiotic conjugates are still potent enough to work as chemical tools to visualize their molecular targets. Examples are the glycopeptide antibiotic vancomycin and the glycolipodepsipeptide ramoplanin that can be used to visualize peptidoglycan biosynthesis in Gram-positive bacteria [64], the polypeptide polymyxin B which targets lipid components of the outer membrane [65] or ribosome-targeting macrolide antibiotics such as erythromycin [66,67,68]. Fluorescent antibiotic conjugates can be used to study cellular permeability to antibiotics [69], cellular and tissue distribution [70] high-throughput drug screening [66,67,68], the antibiotic mode of action (MOA) [71], or to detect and differentiate groups of bacteria [72]. It should be noted that the choice of fluorophore can have a large influence on the biological activity of the resulting probe as was observed when comparing vancomycin-conjugates with the negatively charged fluorescein and those conjugates with the neutral BODIPY [64]. For example, the anionic teichoic acids in the cell wall of Gram-positive bacteria have a repellent effect on negatively charged molecules, allowing uncharged BODIPY-conjugated probes to be taken up more efficiently through the PG layers of the cell wall of such bacteria [64].

Non-covalently targeted fluorescent conjugates are also used to probe specific biomolecular uptake pathways. In contrast to dyes used to study non-specific permeability, uptake probes are conjugates of biomolecular scaffolds that are taken up through interaction with receptors that are part of a specific molecular uptake machinery (e.g., siderophores [25,60] or sugars [73]) that coordinate the selective translocation of the biomolecule (and the functionalized biomolecule-conjugate) through an otherwise largely impermeable cell envelope. This allows accumulation of the probes in cells that possess an active uptake machinery and a differentiation from cells that do not.

#### 2.2.3. Activity-Based Probes

Activity-based probes (ABPs) are functionalized enzyme inhibitors that are designed to interrogate enzyme activity levels in biological samples. The ABPs can rapidly and irreversibly bind to catalytically active target enzymes by selectively and covalently modifying active site residues of the enzyme (Figure 1C). The ABPs basically comprises of three elements: (i) reactive group (also called ‘warhead’), usually an electrophilic group that covalently bind with a conserved active site nucleophile; (ii) linker region or binding group that can modulate the reactivity and specificity of the probe profile [74,75]; (iii) reporter tag for the identification, enrichment and/or visualization of labeled (as reviewed in [74,76,77,78,79]). The modular nature of ABPs in combination with their covalent interaction with probe targets, makes ABPs one of the most versatile group of chemical probes. Usually the same probe scaffold can be used for both visualization as well as pull-down and enrichment of their targets depending on the incorporated functional handle. As a consequence, the range of applications of ABPs is very broad from chemical proteomics identification of enzyme activities and drug targets to non-invasive in vivo imaging of enzymatic activities.

ABPs with different reactive electrophilic groups (e.g., fluorophosphonates [80], chloroisocoumarins [7] carbamates [81], β-lactams [82], β-lactones [82], triazole ureas [83,84,85], Michael acceptors [86], cyclophellitol epoxides and aziridine (see review [87]), disulfides and sulfonate esters [88]) have been developed to selectively study diverse enzyme families including serine hydrolases [7,74,80,89,90], cysteine proteases [91,92], kinases, metalloproteases [93], glutathione-S-transferases [94], cytochrome P450s [95], ATP-binding enzymes [96], or retaining glycosidases [87]. As discussed in more detail below, the most prominent ABP-targets in bacteria are penicillin-binding proteins [97,98], but the use of both broad-spectrum and target-selective ABPs for other bacterial enzymes in both imaging-related [12,84] and proteomic applications [90,99] is emerging.

#### 2.2.4. Substrate Probes

Substrate probes require enzymatic processing to emit a detectable fluorescent or chemiluminescent signal (Figure 1D). In addition to a masked fluorophore or luminophore, a substrate probe must contain a specific chemical group (e.g., an ester or peptide bond) that is actively modified by enzyme targets (e.g., an esterase or protease) and that is part of a larger recognition element that may discriminate between different members of the same enzyme family. These recognition elements include e.g., peptide sequences that are processed differently based on the preferences of proteases with different substrate specificity profiles [12], different acyl groups in ester substrates [7,13,30,31,85,100,101,102] and different sugar moieties in glycosidases substrates. One example of the molecular diversity that such probes can cover is a 32-member library of fluorogenic esterase substrates that Johnson and coworkers developed for structure-activity relationship studies [13]. With respect to imaging and detection in the context of cells or other complex biological samples, the range of applications is mostly guided by the selectivity of the probe. Promiscuous substrates that are processed by many different enzymes found in many organisms have been used to differentiate and even purify cells within bacterial populations based on their ‘overall’ metabolic activity: Examples include the commercial esterase substrate calcein AM [103] and the bacterial reductase probe Redox Sensor^TM^ green [104,105]. On the other hand, substrates with very high specificity hold potential to be used for diagnostic purposes, e.g., in the rapid identification or differentiation of pathogens. However, studies aimed to localize the molecular target, may in general be better addressed by the use of ABPs, which covalently bind to the targets, as the spatial resolution of substrate-probe based imaging is limited due to diffusion of the product after cleavage. However, examples of fluorogenic substrates exist that successfully inform on the subcellular localization of targets: Nitro-aryl based substrates that are converted by ubiquitous nitroreductases (NTR) into bright fluorophores allow precise (punctate) localization of cytosolic NTR by super-resolution imaging of live cells [106]. For a recent overview of the manifold fluorogenic substrates that may be employed in live microscopy or in vivo imaging applications in both mammalian and bacterial systems, the reader is referred to a recent review article [107].

#### 2.2.5. Metabolic Labeling

Another strategy that exploits the bacterial enzymatic machinery for visualization and analysis of biological processes is metabolic labeling. This approach employs chemically modified precursors of a macromolecule of interest and relies on the endogenous enzymatic machinery of the bacteria for probe incorporation (Figure 1E) [108,109,110,111]. Metabolic labeling can be used both for the enrichment and identification of labeled species by molecular analytic methods, but within the scope of this article we will focus on application where cellular structures are visualized. This can be achieved with directly detectable fluorophore-conjugated probes, but very often bio-orthogonal probes are used that are detected in a subsequent step with a compatible reagent. Bio-orthogonal probes are smaller and chemically more similar to the native substrates and are therefore often incorporated more efficiently into cellular macromolecules compared to analogs functionalized with bulky fluorophores. The use of bio-orthogonal probes is more time-consuming compared to the direct use of fluorescent conjugates, but it also has greater versatility as different detection tags may be added in the detection step of the bio-orthogonal labeling process. In the context of microscopic analysis, most common targets of metabolic labeling strategies are proteins [19] and the macromolecules of the cell envelope such as peptidoglycan [16,112] and other glycopolymers [15,113].

#### 2.2.6. Environmental Sensors

In environmental sensors, the properties of the detection group are altered in a certain molecular environment (e.g., in a hydrophobic environment [63] or at a certain pH [52]) or upon reaction with a specific metabolite of interest (e.g., ions [114] or reactive molecules such as formaldehyde [48]), leading to an increase in fluorescence emission or chemiluminescence (Figure 1F).

### 2.3. The Frontier of Chemical Probe Synthesis

Access to chemical probes typically relies on traditional multistep organic synthesis. While this approach has produced a great number of probes, the development of new chemical routes can be a slow and costly process that requires specialist expertise. However, recent developments in the field are paving the way for much more rapid access to new probe designs: Late-stage functionalization is an increasingly popular approach, which aims to alter the chemical structure of already complex molecules (e.g., by directly installing functional groups), thereby greatly reducing the required number of steps needed to synthesize a functionalized analogue of the molecule [115]. This strategy has been made possible by the development of new synthetic methodologies operating under mild reaction conditions, such as photoredox catalysis [116] and C-H activation [117].

A late-stage functionalization approach can for example enable the direct modification of fluorescent probes, and thereby tune, e.g., absorption/emission or solubility properties [118]. Another tantalizing prospect is the direct installation of chemical handles for click chemistry onto drugs or other bioactive molecules [119], which can then be visualized through subsequent bio-orthogonal conjugation with a fluorophore or other observable feature; thus, the strategy rapidly generates a new chemical probe [120]. Indeed, click chemistry itself (including SuFEx chemistry) is emerging not only as a means of bio-orthogonal probe visualization, but also as a tool in the synthesis and late-stage functionalization of new biological probes [18,121,122,123].

## 3. Chemical Probes in Action: Applications in Imaging of Bacteria

**Figure 2:**
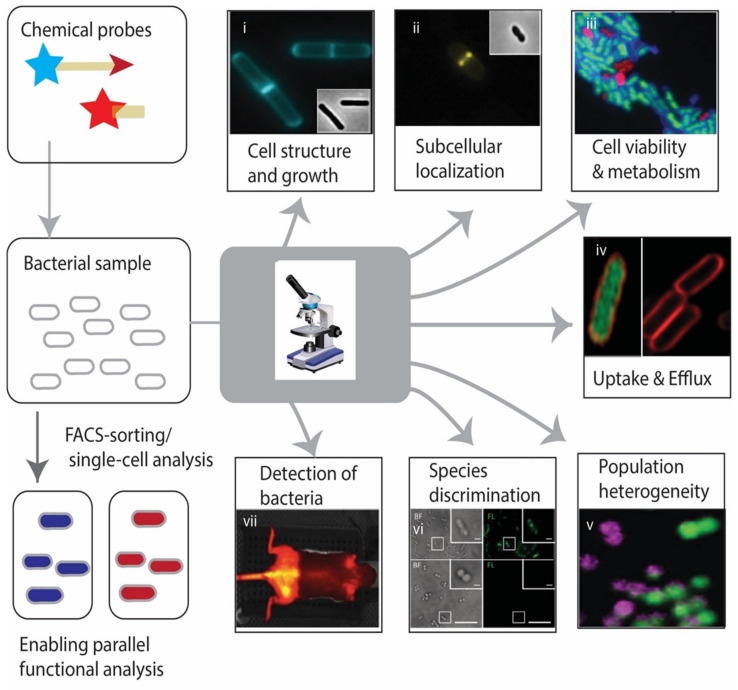
Applications of chemical probes in bacterial imaging. The images demonstrates the utility in difficult probes for visualizing and potential for purification, isolation and downstream analysis by cell-sorting. Exemplary imaging applications are illustrated with images from primary publications: (**i**) Uniform labeling of PG in live *B. subtilis* cells using FDAAs (HADA) [112]. The figure is reproduced with permission from [112]. (**ii**) The subcellular localization of PBPs during cell division and elongation in *Lactococcus lactis* is depicted by Bocillin-FL labeling [130]. The figure is reproduced from [130] under a Creative Commons Attribution (CC BY) license. (**iii**) Live–dead labeling in *E. coli* demonstrates FDAAs (HADA) labeling in live cells (blue) but not dead cells (red) [112]. The figure is reproduced with permission from [112]. (**iv**) Internalization of ciprofloxacin fluorophore derivatives in live *E. coli* with (green, left) and without (red, right) efflux pump inhibitor [126]. The figure is reproduced under a Creative Commons Attribution 3.0 Unported License from [126]—Published by The Royal Society of Chemistry. (**v**) Absence of acticity-based probes (ABP) labeling (purple) in some GFP expressing *S. aureus* cells (green) during exponential phase indicates phenotypically distinct subpopulations [7]. The figure is reproduced with permission from [7]. (**vi**) Vibrios are selectively labeled by the vibroferrin-derived fluorescent siderophore conjugate vibrioferrin-fluorescein (VF-FL) (top) while other species are not labeled (bottom) [60]. The figure is adapted with permission from [60]. Copyright (2017) American Chemical Society. (**vii**) Non-invasive optical in vivo imaging of a mouse with *E. coli* and *S. aureus*-induced myositis in the limb using fluorescently labeled vancomycin [146].

In the following we will highlight some of the most relevant application areas of chemical probe-based imaging. These applications are summarized in Figure 2 and the probes are listed in Table 1.

### 3.1. The Cell Wall

The bacterial cell wall is a complex macromolecular heteropolymer that provides stability to the cell, that controls uptake and release of molecules and that serves as a template for interactions with the environment. Studying the bacterial cell wall is not only relevant to understand cellular morphology, cell division and growth. As interference with cell wall structure and biosynthesis usually has detrimental consequences for the cell, a comprehensive understanding of mechanistic features of the PG structure, function, and biosynthesis is also crucial for the development of novel antibiotics.

The bacterial cell wall contains a rigid layer of peptidoglycan (PG). PG is composed of a glycan chain of repetitive disaccharide units of *N*-acetylglucosamine and *N*-acetylmuramic acid that are crosslinked via peptide bridges most commonly containing L-Ala, D-Ala, D-Glu, Gly, L-Lys, mesodiaminopimelic acid (DAP), or other amino acids. The exact composition and cross-linking architecture of the peptide bridges varies across different families of bacteria (e.g., Gram-positive, Gram-negative and mycobacteria), but also depending on the exact species and based on culture conditions [147,148].

The biosynthesis of peptidoglycan starts in the cytoplasm where the precursors of the disaccharide building blocks UDP-*N*-acetylmuramyl (UDP-Mur*N*Ac)-pentapetide and UDP-*N*-acetylglucosamine (UDP-Glc*N*Ac) are synthesized, attached and combined to a membrane-localized isoprenoid carrier to give the central PG-precursor molecule Lipid II (reviewed in [149]). Lipid II is then flipped to the outer side of the cytoplasmic membrane (in Gram-positive bacteria), which is equivalent to the periplasmic side of the inner membrane of Gram-negative bacteria. Subsequently, the disaccharide units are incorporated into the existing, highly crosslinked peptidoglycan structure through the glycosyltransferase or transpeptidase activity of penicillin-binding proteins (PBPs) (reviewed by [150,151]). DD-transpeptidase activity of PBPs provides peptide cross-linking between D-Ala and DAP, whereas LD-transpeptidases perform cross-link two DAP residues [152]. Additionally, PBPs may also have DD-carboxypeptidase and/or endopeptidase activity [153,154]. Cell wall biosynthesis can be probed and visualized by two main strategies: First, by targeting the enzymatic activities, that shape the cell wall and second, by metabolic labeling of cell wall polymers.

#### 3.1.1. Metabolic Labeling of Peptidoglycan

PG synthesis pathways and cell wall growth and structure can be studied using metabolic labeling with functionalized D-amino acids. Among the most commonly used D-amino acid (DAA) probes are clickable analogs of D-Ala and fluorescent D-amino acid (FDAA) analogs of D-Ala and D-Lys [112] each of which have different incorporation characteristics in different bacterial species. In order to facilitate sequential labeling protocols and ensure compatibility with other staining procedures, FDAAs are available such as blue light emitting (HCC-amino-d-alanine, HADA), green (NBD-amino-d-alanine, NADA, and fluorescein-d-lysine, FDL) or red (TAMRA-d-lysine, TDL) for PG labeling of live bacteria [140]. The use of FDAA has enabled, e.g., the detection of sites of new PG synthesis in cells live bacterial cells, thus distinguishing ‘old’ from ‘new’ PG and also differentiating metabolically active from inactive cells [140]. It has also helped to identify cell division inhibitors, to validate the role of cell division/elongation factors and other factors involved in PG synthesis using mutant strains [140]. Another breakthrough discovery was enabled through clickable D-Ala probes: They have solved the riddle of the ‘Chlamydia anomaly’ (i.e., the fact that *C. trachomatis* is sensitive to PG-targeting antibiotics while no PG could be detected), by visualizing functional PG in *C. trachomatis* for the first time [141].

The incorporation of DAAs into PG can follow three different routes, depending on the species studied: Incorporation can occur into D-Ala-D-Ala through activity of cytoplasmic D-alanine D-alanine ligase (Ddl) or through extracytoplasmatic (e.g., periplasm in Gram-negative bacteria) L,D- or D,D transpeptidases (reviewed in detail in [150,151]). Interestingly, FDAAs were shown to be incorporated into PG of *E. coli* and *B. subtilis* by the extracytoplasmic pathways and not via the intracellular precursor D-Ala-D-Ala [142]. Importantly, the interplay of different routes of PG incorporation is of high relevance for antibiotic activity and resistance as it has been reported that the capability of LD-transpeptidases to bypass DD-transpeptidase activity results in higher level of resistance β-lactam antibiotics in both *E. coli* and *E. faecium* [155,156]. For a more detailed overview on research on how DAA-probes have contributed to our understanding of the dynamics of bacterial growth and division the reader is referred to this excellent review article [157]. A notable chemical innovation for D-amino acid probes was the development of fluorogenic D-amino acids, which provide lower background and are suitable for real-time imaging of peptidoglycan synthesis [8].

#### 3.1.2. Dissecting the Activity of Penicillin-Binding Proteins

Elucidating the specific roles of different PBPs in PG synthesis is important in understanding cell growth and cell wall homeostasis as well as in understanding efficacy and resistance of PBP-targeting antibiotics. Fluorescent penicillin-derivatives (BOCILLIN-FL) have been the first PBP-targeting ABPs. They have been and still are useful tools for detection of PBPs in membrane fractions of labeled cells [129]. BOCILLIN-FL does not discriminate between different members of the PBP family, whereas fluorescently labeled analogs of the beta-lactam antibiotics cephalosporin C and meroponem, as well as beta-lactones have been used successfully to visualize the activity of individual PBPs in the context of living cells [91,131,132].

#### 3.1.3. Targeting Cell Wall Precursors

Another strategy to interrogate PG structure and biosynthesis is by targeting the Lipid II precursor with fluorescent conjugates of vancomycin (a glycopeptide) and ramoplanin (a glycolipodepsipeptide) [64]. In non-invasive in vivo imaging studies, near-infrared fluorescent vancomycin-conjugates have been used to specifically detect murine infections caused by Gram-positive bacteria [146]. In their ability to differentiate Gram-positive from Gram-negative pathogens in complex living animals in real time fluorescent vancomycin-conjugates can be regarded as the ‘Gram stain’ of the 21st century.

Other fluorescent conjugates have been used efficiently to study their mode of action, such as dansylated polymyxins that reveal its interaction with LPS in the outer membrane of Gram-negative bacteria [65,158].

#### 3.1.4. Targeting Other Components of the Cell Envelope

The PG layer also represents anchor sites for the attachment of further macromolecules, including cell wall anchored proteins, anionic polymers such as wall teichoic acid and polysaccharide capsules. In Gram-negative bacteria, surface-exposed polymers such as lipopolysaccharide can be anchored in the outer membrane. 3-deoxy-D-manno-octulosonic acid (KDO) is an essential component of LPS inner core and thus became an attractive candidate for the metabolic probe labeling approach [143]. Another good reason that KDO has been selected as a probe target because the ’clickable’ probe analogue of KDO, such as 8-azido-8-deoxy-KDO is well-tolerated by KDO pathway in a wide range of Gram-negative bacteria [143]. Metabolic incorporation of KDO analogue into bacterial lipopolysaccharides has been shown to be independent of genetic modifications resulting in an efficient tool to investigate LPS structure and their role in the pathophysiological process [143]. In contrast, no broadly applicable technique is available for labeling of teichoic acids in Gram-positive bacteria. However, the unique feature of *S. pneumoniae* to incorporate choline into teichoic acid has enabled the use of propargyl-choline as a bio-orthogonal probe for metabolic labeling of teichoic acids that can be detected via CuAAC [139].

Secreted proteins can be anchored to the bacterial cell wall through the action of transpeptidases that recognize specific peptidic recognition motifs. In *S. aureus*, Sortase A recognizes its substrates based on an N-terminal LPXTG-sequence, cleaves between Thr and Gly and transfers the N-terminal part of the protein onto the free NH_2_-termini in Lipid II [134]. This Sortase-targeting anchorage motif has been exploited for the development of fluorescent and bio-orthogonal chemical probes that are decorated on the bacterial cell wall, enabling the subcellular localization of Sortase A substrates and allows for other cell wall engineering strategies [134,135]. Other cell surface structures such as flagella and pili play a crucial role in the bacterial pathogenesis involving cell motility, adhesion, chemotaxis, and conjugation. To the best of our knowledge, no specific chemical probes have been developed to investigate their function, however, some commercial non-specific fluorescent dyes have been used to capture flagella and pili in action in several bacterial species [9,159,160,161,162].

#### 3.1.5. Trehalose and the Unique Cell Envelope of Mycobacteria

Mycobacteria, which include *M. tuberculosis* (Mtb), have a cell envelope that is very distinct from both Gram-positive and Gram-negative and which includes a unique mycomembrane layer comprising arabinogalactan and long-chain mycolic acids enriched with trehalose-containing glycolipids (reviewed by [163,164]). The application of fluorescent trehalose-conjugates revealed labeling of the mycobacterial membrane and poles and allowed the selective detection of Mtb within macrophages [113]. Other studies employed azide-modified trehalose analogues to visualize cell-surface glycolipids revealing mechanistic insights into the molecular pathways of trehalose modifications and recycling [15]. Trehalose-metabolism has also been exploited to develop an environmentally sensitive fluorescent trehalose probe that lights up within the hydrophobic environment of the mycobacterial cell envelope and can be used to rapidly and sensitively detect Mtb [63]. Intriguingly, a recent study using fluorescent aminoglycoside antibiotics revealed subpopulations of *E. coli* with different probe labeling properties. Weak labeling of a subpopulation was attributed to non-specific membrane-binding of the probe, while cells with high probe-labeling take up the antibiotic in an energy-dependent process, suggesting these two subpopulations possess different susceptibility to aminoglycoside antibiotics [125].

### 3.2. Dissecting Antibiotic Susceptibility and Resistance

Antibiotic resistance can be based on different mechanisms that may be studied using chemical probes, including target bypass mechanisms, antibiotic-modifying enzymes or altered cellular penetration/efflux properties. For PBP, in *S. aureus* or *E. faecium* individual PBP-s have been identified that are associated with low-affinity. With an expanding toolset of ABPs that selectively target PBPs [97,131,132], the visualization of resistance properties using chemical probes appears within reach.

Another reason for β-lactam-resistance may be β-lactamase enzymes that hydrolyse and inactivate β-lactam antibiotics [137]. Fluorogenic umbelliferone-cephalosporin conjugates that serve as a useful tool to study the naturally occurring β-lactamase of Mtb (Bla) [136,137]. It has been demonstrated that cephalosporin based fluorogenic probe is highly selective for Bla and thus applicable for point-of-care diagnostic purposes [136].

Another important mechanism of antibiotic resistance is altered antibiotic uptake and efflux properties due to the reduced expression of porins or increased expression of efflux pumps (detail reviewed [165]). These mechanisms can be studied using fluorescent antibiotic conjugates. Linezolid is a bacterial protein synthesis inhibitor that belongs to the class of oxazolidinone antibiotics and azide-functionalized linezolid probes can be used to visualize AB-uptake into Gram-positive bacteria [72], while the activity of efflux pumps prevents the labeling of Gram-negative cells with this probe [166]. Fluoroquinolone-derived and trimethoprim-derived probes have also been used to study penetration and efflux of bacterial cells and mutant and chemical inhibition studies showed that their intracellular accumulation is dependent on the activity of efflux systems [126,167]. In addition to studying the accumulation of antibiotics in bacterial cells, fluorescent conjugates have also been employed to study the tissue distribution of antibiotics [72].

### 3.3. Visualizing Specific Metabolic Uptake Pathways

#### 3.3.1. Siderophores

Iron is an essential element for bacterial growth and survival. Siderophores secreted and utilized by microbes, engage in scavenging iron from their surroundings, which is crucial for bacterial survival under iron-limiting conditions. In the quest for new and improved drugs, AB-conjugates are an emerging strategy that promises to deliver ABs to cells that are otherwise not AB-sensitive [25,168,169]. These efforts have led to FDA-approval of the siderophore-cephalosporin conjugate cefiderocol which is highly effective against Gram-negative bacteria [170]. As these AB-conjugates turn the pathogen’s own uptake machinery against itself, the approach has been termed Trojan Horse strategy. Siderophore-uptake in *E. coli* is an active, energy-dependent process initiated through binding to receptor proteins on the outer membranes (OM) [171]. Fluorescent and radiolabeled catechol-based siderophore conjugates are efficiently taken up by a variety of bacterial pathogens and have been used for non-invasive optical in vivo imaging of a mouse model of *P. aeruginosa* infection [25]. The authors note that in many instances, they observed in microscopic and flow cytometry experiments that—for unknown molecular reasons—only a subpopulation of cells were able to take up these probes [25], which highlights the utility of chemical probe-based in dissecting functional heterogeneity among cellular populations.

Other siderophore-probes rely on very specific uptake pathways and can only be taken up by a small group of bacteria, allowing for differentiation of bacterial species. One such example is Vibrioferrin (VF), fluorescent conjugates of which fluorescent VF-conjugates can selectively label *Vibrio* species under iron-limiting conditions, allowing their discrimination from *S. aureus* or *E. coli* [60].

#### 3.3.2. Sugar Uptake

Another group of biomolecules whose cellular uptake is driven through a specific molecular machinery are sugars such as maltodextrin. Active maltodextrin uptake involves translocation through the OM via maltoporins, followed by periplasmic binding to maltose-binding protein and translocation through the inner membrane via maltodextrin transporters [124]. Due to a lack of maltodextrin transporters in mammals, fluorescent maltodextrin conjugates can serve as selective tools for non-invasive optical in vivo imaging of bacterial infections [73]. The same group demonstrated in a follow-up study that maltodextrin uptake can also be targeted by radiolabeled probes for visualization of sites of bacterial infections in mice through PET-imaging [26].

### 3.4. Visualizing Virulence-Associated Enzymes

Enzymes can be important virulence factors and chemical probe-based imaging can provide important insight into the subcellular localization and dynamic activity patterns of virulence-associated enzymes. Our recent work on the previously uncharacterized *S. aureus* fluorophosphonate-binding hydrolases (Fph) illustrates how ABPs can be instrumental both in the identification of new enzymatic activities, as well as in their functional validation [7,84]. Fluorescent conjugates of newly identified FphB-specific inhibitors revealed selective labeling of the target enzyme at specific sites of the cell envelope and growth-condition dependent labeling of the septal cross wall of dividing cells, suggesting its physiological role may be modification of substrates located in the cell wall [7,84]. We also demonstrated that FACS-analysis of ABP-labeled bacterial populations is suitable to dissect the dynamic distribution of enzymatic activity levels across cells in different growth environments [7,84].

In other instances, virulence factors have been targeted to enable probe-based pathogen-specific detection, which is the case for the cell envelope-associated *M. tuberculosis* protease Hydrolase-important for pathogenesis 1 (Hip 1) [12]. Elucidation of the proteolytic substrate selectivity profile of Hip1 has enabled the generation of highly specific hybrid canonical/non-canonical peptide fluorogenic substrates that are turned over by Mtb in a Hip1-dependent manner and promise the development of Mtb-specific imaging agents [12]. This Hip1-targeting peptide sequence has recently been introduced into the luminogenic probe FLASH that detects Hip1 activity with extremely high sensitivity [45].

Secreted nucleases are important virulence factors of *S. aureus* that are involved in degrading extracellular DNA of *S. aureus* biofilms. Quenched fluorescent oligonucleotide substrates with chemical modifications to prevent cleavage by mammalian nucleases provide a *S. aureus* nuclease-specific signal that has enabled non-invasive in vivo imaging of *S. aureus* infections in a mouse pyomyositis model [138]. Similar FRET-based nuclease probes were also useful to image surface-localization of the nuclease Nuc2 activity and identified peak activity during early logarithmic growth [172].

### 3.5. Biofilms and Other Microbial Communities

Biofilms are heterogeneous bacterial communities that are embedded in a self-produced extracellular matrix of polysaccharides, proteins and DNA. Biofilms are most often surface-associated and in the context of medical device/implant-associated infections, they are of great clinical concern as they are difficult to eradicate. For biofilm settings, the use of chemical probes is helpful visualize and quantify functionally different cells within the population. The most simple and common functional differentiation of cells within biofilms is between live and dead cells using a combination of different non-specific dyes with different cellular permeability in live and dead cells [173,174]. It should be noted, however, that factors other than cell death can affect cellular permeability read-outs [5,6].

Another clinically relevant cellular subpopulation of bacteria are persister cells. Persister cells are phenotypic variants within isogenic bacterial populations that can survive antibiotic treatment without developing genetic antibiotic-resistance [175]. These cells are morphologically indistinguishable from the bulk of antibiotic-susceptible cells. Brynildsen and coworkers applied fluorogenic substrates that are activated by reductases as markers for metabolic activity in combination to fluorescent-activated cell-sorting (FACS) and persister cell assays. These elegant studies revealed that, in *E. coli*, spontaneously occurring persisters in exponential phase were mostly derived from metabolically-dormant cells (with low fluorogenic probe labeling) [104], whereas the formation of triggered persisters in stationary phase required high redox-activity (high probe labeling) [105].

Another study employed the non-specific esterase probe calcein-AM in mycobacteria, followed by FACS-sorting, and antibiotic susceptibility testing [103]. The authors continued with a FACS-based transposon mutant screen that identified factors that affect calcein-AM staining in cells. This included obvious candidates such as esterases and efflux pumps, but also identified a mycobacterial divisome factor responsible for heterogeneity in polar growth. [103]

Target-selective probes can also serve as reporters for phenotypic heterogeneity in bacterial population as our already mentioned studies on the distribution of the enzymatic activities of the *S. aureus* serine virulence factors FphB and FphE across growth conditions revealed [7,84]. While the physiological relevance and molecular mechanisms behind this observation remain unclear, it showcases how the use of chemical probes in cellular imaging can uncover new biology—both by design and by serendipity.

While the previous examples illustrate the power of chemical probes in dissecting the physiological diversity within populations of single bacterial species, they have also been applied to deconstruct even more complex samples taken from environmental communities or the human microbiome. Whidbey and coworkers used beta-glucuronidase-specific activity-based probes to isolate bacterial subpopulations from mouse fecal sample and determine beta-glucuronidase-active taxa within the gut microbiome [133].

One emerging metabolic labeling strategy that has been used to differentiate single cells of environmental bacterial samples based on their translational activity is ’Bio-orthogonal non-canonical amino acid tagging’ (BONCAT [19,144]). BONCAT makes use of azide-/or alkyne-functionalized analogs of L-methionine, which are incorporated into newly translated proteins and can be visualized by conjugation fluorescent dyes in a bio-orthogonal reaction post-labeling [19] and allows the separation of cells based on their anabolic activity by fluorescence-activated cell sorting [62,144].

## 4. Concluding Remarks and Future Perspectives

Technological innovations have always been pushing the frontiers of (micro)biological research, but the bleak reality of the emerging antimicrobial resistance (AMR) crisis forces us to revisit research topics that were nearly considered done with decades ago: The development of new and improved antimicrobial treatment strategies is back on top of the research agenda of the (medical) microbiology community. One potential future scenario to control the AMR crisis relies on the development of sustainable personalized medicine strategies that break the vicious circle of antibiotic exposure and resistance development: targeted narrow-spectrum treatment strategies that prevent pathogenesis or increase antibiotic susceptibility by modulating bacterial physiology rather than killing them. For such a scenario to become reality, it is necessary to improve understanding of bacterial physiology, virulence and AMR within the host and translate this knowledge to develop new precision chemical tools to manipulate bacterial physiology and as point-of-care diagnostic tools. Chemical probes can provide such high-level biological validation of target-engagement or drug uptake in increasingly complex physiological environments. Furthermore, thanks to the continuous improvement of reporter fluorophores and luminophores, chemical probes have great potential to yield sufficient sensitivity and specificity to serve as diagnostic tools for in vivo and ex vivo assessment of clinically relevant parameters such as AMR and virulence to inform treatment strategies.

The single-cell frontier and the assessment of cellular phenotypic heterogeneity is another area where chemical probes are instrumental [79]. Bacterial biofilms, antibiotic persistence and the elusive viable-but-non-culturable bacteria are examples based on the presence of functionally distinct subpopulations within pathogen populations. Yet, most traditional ’phenotypic assays’ and—more recently—‘omics’-based approaches that describe functional responses that were commonly done in bulk populations provide a functional read-out of the average cell as an insufficient approximation of the more complex, heterogenous reality. Again, thanks to biotechnological advances, this is changing and a recent breakthrough has been the elucidation of growth-condition specific transcriptomes of single bacteria [176]. Yet without the introduction of exogenous labels, phenotypically distinct subpopulations remain morphologically indistinguishable from each other. Hatzenpichler and coworkers recently coined the term next-generation physiology to describe a set of experimental approaches that pair non-destructive phenotypic analysis of single cells within complex microbiome samples with cell isolation techniques and downstream analysis [145]. Although, label-free phenotypic analyses are possible in this context, chemical probe-based differentiation of cellular phenotypes is a cornerstone of such experimental strategies. In contrast to biological reporters, which require genetic manipulation and are limited to a strain of interest, chemical probes are universally applicable to any native sample of interest (as far as its specificity and permeability profile allow), provide a phenotypic read-out and may serve as a biomarker for the isolation of single cells or subpopulations of interest. Furthermore, in times where the limits of multiplexed cellular fluorescence analysis are being pushed through advances in full-spectrum flow cytometry [177], the development of an increasing palette of sensitive fluorophores and fluorogens with non-overlapping excitation-emission spectra promises the simultaneous assessment of numerous phenotypic traits. Another technique for highly multiplexed parallel analysis of chemical probe-labeling of cells is mass cytometry: Poreba and co-workers recently introduced lanthanide-labeling into ABP design allowing mass cytometry-based analysis of four mammalian proteases [178]. Although the technique is merely analytical as the cells are destroyed during the cytometry by time-of-flight application, it may allow the parallel assessment of >20 differently tagged probes.

These developments are further supported by progress along the frontier of synthetic chemistry, where methods are emerging for the facile production of entirely new probes, linkers and bioconjugation strategies, providing a steadily expanding chemical biology toolkit that will allow microbiologists to study and manipulate biological systems with unprecedented versatility and precision.

## Figures and Tables

**Table 1:** Chemical probes and their application in visualizing bacterial structure and physiology.

Probe Name	Probe Type	Targeted Species	Molecular Target	Detection Tag(s)	Application	References
Vibrioferrin-FL	Non-covalent targeted conjugate	*V. parahaemolyticus*, *V. cholerae*, and *V. vulnificus*	Siderophore uptake pathway	Fluorophore/Bio-orthogonal handle	Visualization of vibrioferrin uptake and selective detection of Vibrios under iron-limited conditions	[60]
DOTAM–FL	Non-covalent targeted conjugate	*P. aeruginosa* and *E. coli*	Siderophore uptake pathway	Fluorophore	Visualization of iron transport and detection of bacterial infections	[25]
MDPs	Non-covalent targeted conjugate	*E. coli*, *P. aeruginosa*, *B. subtilis*, and *S. aureus*	Maltodextrin uptake pathway	Fluorophore/Radiolabel	Visualization of maltodextrin uptake and high-sensitivity detection of bacteria *in vivo*	[27,73,124]
Neo–Cy5	Non-covalent targeted conjugate	*P. aeruginosa*, *A.* *baumannii*, *K. pneumoniae*, *S. typhimurium*, and *S. aureus*	Aminoglycoside antibiotics uptake pathway	Fluorophore/Bio-orthogonal handle	Visualization of aminoglycoside uptake and mode of action	[125]
Cipro-azide	Non-covalent targeted conjugate	*E. coli* and *S. aureus*	Antibiotics uptake pathway	Fluorophore/Bio-orthogonal handle	Understanding the bacterial penetration and efflux pump mechanisms	[126]
Van-FL	Non-covalent targeted conjugate	*B. subtilis*, *S. pneumoniae*, *S. coelicolor* and *C. glutamicum*	PG stem peptide (D-Ala-D-ALA)	Fluorophore	Visualize nascent PG biosynthesis in live cells	[64,127]
BOCILLIN-FL	Activity-based probe	*E. coli*, *P. aeruginosa*, and *S. pneumoniae*	Active PBPs (broad spectrum)	Fluorophore	Broad-spectrum detection of PBP activities in live cells.	[128,129,130]
Ceph C-T	Activity-based probe	*B. subtilis* and *S. pneumoniae*	PBPs 1a/1b, 2b, 2c, and 4 (*B. subtilis*) and PBP1b and 3b (*S. pneumoniae*)	Fluorophore	Visualize involvement of different PBP subsets in live cells	[97]
β-lactone probes	Activity-based probe	*S. pneumoniae*	PBP1a, PBP1b, PBP2x, and PBP2a	Fluorophore	Visualize the catalytic activity of PBP subsets in live cells	[131]
Meropenem-derived probe MEM-FL	Activity-based probe	*B. subtilis*	PBP3 and 5	Fluorophore/Bio-orthogonal handle	Visualize PBP3 activity in single cells during cell division	[132]
Fluoro-phosphonates (FP-TMR)	Activity-based probe	*S. aureus*	Serine hydrolases	Fluorophore/Biotin	Identification of serine hydrolase activities	[7,89]
JCP251-bT	Activity-based probe	*S. aureus*	Fluorophos-phonate-binding serine hydrolase B (FphB)	Fluorophore	Visualize subcellular FphB localization and distribution across cell population	[7]
Triazole urea probes	Activity-based probe	*S. aureus*	Fluorophos-phonate-binding serine hydrolases and lipases	Fluorophore/Bio-orthogonal handle	Assessment of specific cellular serine hydrolase activity levels	[84]
GlcA-ABP	Activity-based probe	Mouse gastrointestinal microbes	β-glucuronidase	Fluorophore/Bio-orthogonal handle	Detection, isolation and identification of microbial subpopulations in the gut microbiome	[133]
CSL174	Substrate probe	*M. tuberculosis*	Hydrolase-important for pathogenesis 1 (Hip1)	Fluorophore	Specific detection of Hip1 protease activity	[12]
FLASH	Substrate probe	*M. tuberculosis*	Hydrolase-important for pathogenesis 1 (Hip1)	Chemiluminescent	Detection of live *M. tuberculosis*	[45]
Calcein- AM	Substrate probe	*M. tuberculosis* and *Mycobacterium smegmatis*	Esterases	Fluorophore	Single-cell assessment of esterase activity and probe uptake	[103]
Redox Sensor Green (RSG)	Substrate probe	*E. coli*	Bacterial reductase	Fluorophore	Assessment of cellular redox activity	[104,105]
LPETG-derived peptides	Metabolic labeling	*S. aureus*	Sortase A–dependent cell wall anchoring	Fluorophore/Bio-orthogonal handle	Imaging of cellular Sortase A levels, cell wall re-engineering	[134,135]
CLSP and CLLP	Substrate probe	*Salmonella* spp. and *L.* *monocytogenes*	Esterase and phosphatidylinositol-specific phospholipase C (PI-PLC)	Luminophore	Selective detection of *Salmonella* spp. and *L.monocytogenes* from food samples	[44]
Nitro-aryl fluorogen	Substrate probe	*B. subtilis*	Nitroreductase activity	Fluorophore	Visualization subcellular localization of nitroreductases	[106]
CDG-OMe	Substrate probe	*M. tuberculosis*	β-lactamase (Bla) C	Fluorophore	Detection of live *M. tuberculosis*	[136,137]
Cy5. 5-TT	Substrate probe	*S. aureus*	Micrococcal nuclease (MN)	Quenched fluorophore	Noninvasive detection of *S. aureus* infections in mouse pyomyositis model	[138]
D-alanine analogues	Metabolic labeling	*L. monocytogenes*	Peptidoglycan	Bio-orthogonal handle	Visualization of PG dynamics	[16]
Propargyl-choline	Metabolic labeling	*S. pneumoniae*	Teichoic acid	Bio-orthogonal handle	Visualization of pneumococcal teichoic acid biosynthesis.	[139]
FDAA	Metabolic labeling	*B. subtilis*, *E. coli*, *S. aureus*, *S. pneumoniae*, *Agrobacterium tumefaciens* and *C. crescentus*	PG stem peptide	Fluorophore/Bio-orthogonal handle	Visualization of PG biosynthesis and illustration of bacterial growth and division	[61,112,140,141,142]
KDO	Metabolic labeling	*E. coli* and *Salmonella typhimurium*	LPS	Fluorophore/Bio-orthogonal handle	Visualization of LPS structure and location	[143]
Homopropargylglycine (HPG)	Metabolic labeling	Sulfate-reducing bacteria, uncultured microbes	Protein synthesis	Bio-orthogonal handle	Single-cell assessment of translational activity	[144,145]
L-azidohomo-alanine (AHA)	Metabolic labeling	*E. coli*, single environmental bacterial strains and complex samples	Protein synthesis	Bio-orthogonal handle	Single-cell assessment of translational activity	[19]
Azido-modified trehalose	Metabolic labeling	*M. tuberculosis*	Cell surface glycolipids	Bio-orthogonal handle	Detection and visualization of cell-surface glycolipids	[15]
Trehalose analogs	Metabolic labeling	*Mycobacterium* spp	Myco-membrane	Fluorophore/Bio-orthogonal handle	Determination of the envelope structure of *Mycobacterium*	[113]
Thioflavin T (ThT)	Non-specific fluorescent dye	*B. subtilis*	Membrane	Fluorophore	Quantification of membrane potential	[114]
DMN-Tre	Environmental sensor/Metabolic labeling	*M. tuberculosis*	Myco-membrane	Fluorophore	Detection of *M. tuberculosis*	[63]

## Abbreviations

ACC	7-amino-4 carbamoylmethylcoumarin
AM	Acetoxymethyl
BONCAT	Bio-orthogonal non-canonical amino acid tagging
BODIPY	4,4-difluoro-4-bora-3a,4a-diaza-s-indacene
CuAAC	Copper-catalyzed azide-alkyne cycloaddition
Cy5	Cyanine 5
DAA	D-amino acid
DAP	mesodiaminopimelic acid
DDAO	7-hydroxy-9H-(1,3-dichloro-9,9-dimethylacridin-2-one)
Ddl	D-alanine-D-alanine ligase
DNA	Deoxyribonucleic acid
DMN	4-*N*,*N*-Dimethylamino-1,8-naphthalimide
FACS	Fluorescence-activated cell sorting
FDA	Federal drug administration
FDAA	Fluorescent D-Amino Acid
FDL	Fluorescein-D-Lysine
FRET	Fluorescence resonance energy transfer
GFP	Green fluorescent protein
HADA	HCC-amino-D-alanine
Hip1	Hydrolase important for pathogenesis 1
KDO	3-deoxy-D-manno-octulosonic acid
LPS	Lipopolysaccharide
MOA	Mode of action
Mtb	*Mycobacterium tuberculosis*
NADA	NBD-amino-D-alanine
NBD	7-Amino-4-nitro-2,1,3-benzoxadiazole
NTR	nitroreductase
OM	Outer membrane
Fph	Fluorophosphonate-binding hydrolase
PBP	Penicillin-binding proteins
pNA	p-nitroaniline
PET	Positron-emission tomography
PG	Peptidoglycan
SDS-PAGE	Sodium dodecyl sulphate-polyacrylamide gel electrophoresis
STORM	Stochastic optical reconstruction microscopy
SuFEx	Sulfur fluoride exchange
TAMRA	Tetramethylrhodamine
TDL	TAMRA-d-lysine
VF-FL	Vibrioferrin-Fluorescein
